# The prognostic value of a laboratory cachexia score (LCAS) defined by LDH, CRP and albumin in patients with advanced lung cancer

**DOI:** 10.1186/s12885-025-13426-3

**Published:** 2025-03-25

**Authors:** Joshua J. Thompson, Nicholas MacLeod, Sarah Will, Fraser O’Rourke, Josh McGovern, Campbell Roxburgh, Joanne Edwards, Ross D. Dolan, Donald C. McMillan

**Affiliations:** 1 Academic Unit of Surgery, University of Glasgow, Glasgow Royal Infirmary, New Lister Building, 10-16 Alexandra Parade, Glasgow, G31 2ER UK; 2 West of Scotland Cancer Centre, The Beatson, 1053 Great Western Road, Glasgow, G12 0YN UK; 3 School of Cancer Sciences, University of Glasgow, Wolfson Wohl Building, Garscube Estate, Switchback Road, Glasgow, G61 1QH UK

**Keywords:** Lung Cancer, Lactate Dehydrogenase, Performance status, Cachexia, GLIM. CRP, mGPS

## Abstract

**Background:**

Systemic inflammation has prognostic value in cancer and is considered aetiological of cachexia by the Global Leadership Initiative on Malnutrition (GLIM). Lactate dehydrogenase (LDH) also has recognized prognostic value. The present study aimed to evaluate the ability of a laboratory cachexia score (LCAS) defined by LDH, CRP and albumin, to identify cachexia and predict outcome in advanced lung cancer.

**Methods:**

Patients (*n* = 261) with serum LDH, CRP and albumin measurement receiving palliative radiotherapy for advanced lung cancer between 2009 and 2015 were identified. Subjects were stratified by LDH and LCAS. This was compared to GRIm and LIPI, two previously described LDH based prognostic scores, which do not incorporate CRP.

**Results:**

On follow up there were 201 deaths. LDH and LCAS were associated with 1-year survival independent of ECOG-PS, MUST, weight loss, BMI, SMI, SMD, metastases, mGPS or NLR (all *p* < 0.001). On multivariate analysis LCAS (1.36, 1.13–1.63, *p* = 0.001), LIPI (1.50, 1.17–1.92, *p* = 0.02), metastases (1.53, 1.15–2.04, *p* = 0.004) and ECOG-PS (1.28, 1.04–1.57, *p* = 0.019) were independently associated with poorer overall survival.

**Conclusion:**

LCAS appears to identify cachexia and stratify survival. This may represent a useful aetiological criterion within the GLIM framework and a more powerful prognostic tool than the phenotypic criteria.

## Introduction

Cancer cachexia is a calamitous syndrome of anorexia, fatigue and weakness accompanied by loss of lean body tissue. The all too often presence in advanced cancer heralds poor prognosis. Frustratingly, the lack of a consensus definition [1–4] and recognized trial endpoints [5] has led to the under diagnosis of cachexia, challenges to trial design and disappointing progress regarding treatment options.

Historically, cachexia was considered a nutritional problem yet increasing credence has been given to metabolic dysfunction and inflammation as both originating and driving forces. Most recently, the Global Leadership Initiative on Malnutrition (GLIM) published a definition of cachexia which centers around disease related malnutrition with inflammation. GLIM recommends the application of a validated screening tool for malnutrition, with the diagnosis confirmed by the presence of both an aetiologic and phenotypic criterion of cachexia [3]. The phenotypic criteria are; involuntary weight loss, low body mass index (BMI) and low muscle mass. The aetiologic criteria are reduced food intake or assimilation and disease burden/inflammation.

GLIM assigns equal value to the phenotypic and aetiologic criteria however work suggests that inflammation dominates the prognostic landscape. Recently, Zhang and co-workers examined the prognostic value of a combined weight loss and inflammation grade (WLAIG) in over 11,000 patients with advanced cancer [6]. They showed this to be a robust marker of prognosis but of note, weight loss was less prevalent than inflammation across all tumor stages, and inflammation had greater prognostic value in weight losing patients than weight loss did in patients who were not inflamed. Similar conclusions have been drawn with regards to skeletal muscle mass [7]. Hacker et al. examined the relationship between systemic inflammation (as measured by mGPS) and skeletal muscle index (SMI) in patients with oesophageal and gastric cancer [8]. They demonstrated that systemic inflammation was associated with a low SMI but that inflammation was the dominant prognostic factor and suggested there was no direct link between sarcopenia and survival. A recent review by McGovern et al. describes how recent evolution in the cachexia definition has moved systemic inflammation to the forefront [9] and propose that cachexia be considered as disease related inflammation with malnutrition.

Metabolic dysfunction is another central component of tumour biology with lactate dehydrogenase (LDH) shown to have prognostic value in cancer [10]. A constituent enzyme from the oxidoreductase class, it catalyzes the conversion of pyruvate to lactate via the reduction of NAD^+^ to NADH in both directions [11]. As such, it is integral to glucose metabolism under anaerobic conditions. The consequences of this pathway are the inefficient production of adenosine triphosphate (ATP) and generation of lactate, a metabolic dead end which reduces the pH of the surrounding environment. Interestingly, tumour cells often metabolize glucose in this manner, despite the presence of oxygen, an observation termed the “Warburg effect” [10, 12, 13].

Recently, the relationship between LDH and the GLIM criteria was examined in a prospective cohort of patients with advanced cancer. LDH was associated with performance status, systemic inflammation and survival but not weight loss, BMI or reduced skeletal muscle (the phenotypic GLIM criteria) [14]. Furthermore, a recent meta-analysis and systematic review examined the prognostic value of, and associations between, LDH and the GLIM criteria [15]. In over 40,000 patients with cancer LDH was as prognostic of overall survival as any of the GLIM criteria and was associated with the systemic inflammatory response as measured by CRP, the modified Glasgow Prognostic Score (mGPS) and neutrophil-lymphocyte ratio (NLR). There was a weak association between LDH and low BMI but no association with weight loss or low skeletal muscle mass [15].

The prognostic value of inflammation as measured by the mGPS has been widely validated in cancer over the last 20 years. Although prognostic scores of metabolic function incorporating LDH have been described, none have been validated on the same scale. The Royal Marsden Hospital (RMH) score was the first of these to be described. As Phase I trials are designed to assess the tolerability and toxicity of new therapies, they require patients with good performance status, good organ function and a life -expectancy of at least 3 months. This can be challenging to predict. The RMH score which consisted of LDH, albumin and number of metastatic sites was considered to stratify prognosis in these patients. Further scores including LDH were described, but of particular interest are two which also incorporate a measure of inflammation. The Gustave Rousy Immune (GRIm) score and Lung Immune Prognostic Index (LIPI) are both LDH centered prognostic scores which also incorporate the neutrophil-lymphocyte ratio (NLR) as a measure of systemic inflammation (Table 1).

There is extensive evidence that systemic inflammation and metabolic dysfunction are both at play in driving cancer cachexia. How these observations interact remains unclear and to our knowledge there is no published work examining the prognostic value of the presence of both inflammation as measured by mGPS and metabolic dysfunction as measured by LDH. The authors propose an objectively measured laboratory cachexia score (LCAS) which combines systemic inflammation as measured by the widely validated mGPS [16–18] (a CRP and albumin-based score) and metabolic dysfunction as measured by LDH.

The aim was to evaluate the ability of LCAS to both identify cachexia and predict outcome in a retrospective cohort of advanced lung cancer patients receiving palliative radiotherapy, a patient population known to have a high prevalence of cachexia. Its prognostic value was also compared to that of the Malnutrition Universal Screening Tool (MUST), the GLIM criteria for cancer cachexia and the previously reported LDH and NLR based GRIm and LIPI scores.

## Patients and methods

Patients receiving palliative radiotherapy for advanced lung cancer at The Beatson West of Scotland Cancer Institute between 2009 and 2015 were retrospectively identified. Patients were considered eligible if they received palliative radiotherapy and were above 18 years of age, with advanced lung cancer (defined as stage III or IV disease), of any pathological sub-type, and had a measured serum LDH within 6 weeks prior to radiotherapy. Patients were not excluded if they had received prior radical anti-cancer treatment. Point of study entry was taken as the date of serum LDH measurement. This study conformed to Declaration of Helsinki and was approved by Health Research Authority Ethics Committee (17/NW/0190) of Greater Glasgow and Clyde NHS Health Board. Due to the retrospective nature of the study informed consent was either impossible or impracticable, however this was acknowledged within the above ethical approval. The study conformed to the Strengthening the Reporting of Observational Studies in Epidemiology (STROBE) guidelines [19].

Baseline demographic and clinicopathological variables were gathered for each patient (Table 2.). As GLIM recommend the use of a validated malnutrition screening tool, Malnutrition Universal Screening Tool (MUST) score was calculated as this was the locally used tool [20]. Eastern Co-operative Oncology Group Performance Status (ECOG-PS) was recorded if this had been documented by the patient’s oncologist or respiratory physician. ECOG-PS was then grouped as 0–1/2/3–4, as previously described [21]. Serum LDH values were taken from serum blood samples. Any LDH value determined from pleural fluid was excluded. LDH was dichotomized to normal or raised with a cut off value of *≥* 250 Units/L. The primary outcome of interest was overall survival at 1 year from the date of serum LDH measurement.

### Laboratory cachexia score (LCAS)

LCAS was constructed by combining mGPS and LDH. An mGPS score of 0/1/2 is determined by the presence of a CRP < or *≥* 10 mg/L and an Albumin < or *≥* 35 g/L. Lactate dehydrogenase was incorporated in an additive fashion by allocating a score of 0 or 1 based on a threshold of < or *≥* 250 Units/L. This gives a potential LCAS score of 0/1/2/3 as outlined in Table 1.

**Table 1:** Calculation of a laboratory cachexia score (LCAS), Gustave Rousy Immune score (GRIm) and lung Immune Prognostic Index (LIPI). LDH = lactate dehydrogenase, mGPS = modified Glasgow Prognostic score, NLR = neutrophil lymphocyte ratio and dNLR = derived neutrophil lymphocyte ratio

**LCAS**	Points Allocated
LDH < 250 Units/L and mGPS = 0	0
LDH < 250 Units/L and mGPS = 1	1
LDH ≥ 250 Units/L and mGPS = 0	1
LDH ≥ 250 Units/L and mGPS = 1	2
LDH < 250 Units/L and mGPS = 2	2
LDH ≥ 250 Units/L and mGPS = 2	3
**GRIm**	
LDH ≤ ULN and Albumin ≥ 35g/L and NLR ≤ 6	0
LDH > ULN and Albumin ≥ 35g/L and NLR ≤ 6	1
LDH ≤ ULN and Albumin < 35g/L and NLR ≤ 6	1
LDH ≤ ULN and Albumin ≥ 35g/L and NLR > 6	1
LDH > ULN and Albumin < 35g/L and NLR ≤ 6	2
LDH > ULN and Albumin ≥ 35g/L and NLR > 6	2
LDH ≤ ULN and Albumin < 35g/L and NLR > 6	2
LDH > ULN and Albumin < 35g/L and NLR > 6	3
**LIPI**	
LDH ≤ ULN and dNLR ≤ 3	0
LDH > ULN and dNLR ≤ 3	1
LDH ≤ ULN and dNLR > 3	1
LDH > ULN and dNLR > 3	2

### The gustave-rousy immune (GRIm) score

The GRIm score was calculated as previously described [22]. A point is allocated for each of an albumin < 35 g/L, an LDH greater than the upper limit of normal and an NLR of > 6. This results in a score of 0/1/2/3 as outlined in Table 1.

### The lung immune prognostic index (LIPI)

The LIPI score was calculated as previously described [23]. A point is allocated for an LDH greater than the upper limit of normal and a derived NLR > 3 (dNLR = (leucocyte count - neutrophil count)/leucocyte count). This results in a score of 0/1/2 as outlined in Table 1.

### GLIM criterion for diagnosing cancer cachexia

As the most recent recognized consensus on the definition of cachexia, the presence phenotypic and aetiologic GLIM criteria was assessed in each patient. As described above the phenotypic criteria are unintentional weight loss, low BMI and reduced skeletal muscle. Weight loss was assessed as present or absent based on documentation of the attending oncologist or respiratory physician’s clinic letters. The retrospective nature of this study means quantifying percentage weight loss was not possible. A low BMI was considered < 20 kg/m^2^ in patients aged < 70 years and < 22 kg/m^2^ in patients *≥* 70 years. Reduced skeletal muscle mass was defined as a low SMI as described below. The aetiologic criteria are reduced food intake or assimilation and disease burden/inflammation. Food intake was not assessed in this study. The presence of systemic inflammation was determined using the modified Glasgow Prognostic Score (mGPS) and the neutrophil-lymphocyte ratio (NLR) calculated from venous blood samples. NLR was calculated by dividing the neutrophil count by lymphocyte count as is grouped as < 3/3–5/>5 [24]. The mGPS was calculated as described previously and given a value of 0/1/2 [4]. An autoanalyzer was used to measure CRP (mg/L) and albumin (g/L) concentrations according to routine laboratory protocols.

### CT-derived skeletal muscle mass

Reduced skeletal muscle was assessed by analysis of CT scans. Images were taken at the level of the third lumbar vertebra as previously described. These images were then processed using the program Slice-o-matic v.6 (Tomovision, Magog, Canada, /https://www.tomovision.com/products/sliceomatic.html) to produce values for skeletal muscle density and skeletal muscle index.

Definitions for both low SMI and low SMD were those described by Martin et al. [25]. Low SMI (sarcopenia) was defined as < 43cm^2^/m^2^ if BMI *≤* 25 kg/m^2^ or SMI < 53cm^2^/m^2^ if BMI *≥* 25 kg/m^2^ in male patients and an SMI < 41cm^2^/m^2^ regardless of BMI in female patients. Low SMD (myosteatosis) was defined as SMD < 41 Hounsfield Units (HU) and BMI < 25 kg/m^2^ or SMD < 33 HU and BMI *≥* 25 kg/m^2^.

### Statistical analysis

Demographic data, clinicopathological variables, LCAS, LDH, ECOG-PS, weight loss, MUST, BMI, SMI, SMD, NLR, mGPS, 3-month, 6-month and 1-year survival were presented as categorical variables. These were analysed using Chi-squared test for linear-by-linear association.

Demographic data, clinicopathological variables, LCAS, LDH, ECOG-PS, weight loss, MUST, BMI, SMI, SMD, NLR, mGPS and 1-year survival were examined using univariable and multivariable Cox regression analysis to generate univariable and multivariable hazard ratios (HR) for overall survival. Any variable which had a univariable HR with a significance of *p* < 0.05 was included in a backwards conditional multivariable model.

Missing data were excluded from the analysis on a variable-by-variable basis. Two-tailed *p* values < 0.05 were considered statistically significant. All statistical analysis was undertaken in IBM SPSS Statistics Version 29.0.1.0 (101) (SPSS Inc., Chicago, IL, USA).

## Results

A total of 261 patients were included in the analysis. The clinicopathological characteristics of the studied cohort are outlined in Table 2. 51% were male and 70% were *≥* 65 years of age. All patients had advanced lung cancer (*n* = 261), of whom 44% had received palliative chemotherapy at the time of laboratory measures. However, the chemotherapy agents or number of cycles were not available to report. The median LDH was 251 Units/L (102–3815) with 50% of subjects having an LDH *≥* 250 Units/L. 82% of patients had an ECOG-PS of *≥* 1 and 44% had documented weight loss at the time of diagnosis. 176 patients had a recorded BMI, and of these 15% were classified as having low BMI. 53% of patients had reduced muscle mass and 60% had reduced muscle density. 52% of patients had metastatic disease at diagnosis and 77% had nodal spread. NLR was *≥* 3 in 71% of patients and mGPS *≥* 1 in 76%. 17% of patients had an LCAS (Table 1) of 0, 19% an LCAS of 1, 40% an LCAS of 2 and 23% an LCAS of 3. The median survival was 11.1 months (0-150). Survival was 69% at 3 months, 48% at 6 months and 23% at 1 year.

The relationship between LDH and clinicopathological characteristics and survival in patients with advanced lung cancer is shown in Table 2. There was a significant association between LDH and age (*p* = 0.04), chemotherapy (*p* < 0.001), mGPS (*p* = 0.029) and 3-month (*p* < 0.001), 6-month (*p* < 0.001) and 1-year survival (*p* < 0.001).

**Table 2:** The relationship between serum lactate dehydrogenase (LDH) and clinicopathological variables, Eastern Cooperative Oncology Group performance status (ECOG-PS), weight loss, malnutrition universal screening tool (MUST) score,, body mass index (BMI), skeletal muscle index (SMI), skeletal muscle density (SMD), disease burden, systemic inflammation, and survival in advanced lung cancer (*n* = 261)

	LDH < 250 Units/L (*n* = 131)	LDH *≥* 250 Units/L (*n* = 130)	*p*-value^a^
**Age**			*p* = 0.044
< 65	36 (28)	42 (32)	
65–74	41 (32)	54 (41)	
> 74	54 (40)	34 (27)	
**Sex**			*p* = 0.240
Male	72 (55)	62 (48)	
Female	59 (45)	68 (52)	
**ECOG-PS**			
0/1	69 (53)	75 (58)	*p* = 0.557
2	47 (36)	40 (31)	
3/4	14 (11)	14 (11)	
**T-stage**			
3			
4			
**Subtype**			
SCLC	6 (5)	15 (13)	
NSCLC	106 (95)	103 (87)	
Other			
**Metastatic Disease**			*p* = 0.072
Yes	61 (47)	75 (58)	
No	70 (53)	55 (42)	
**Nodal Disease** ^**b**^			*p* = 0.817
Yes	85 (77)	88 (78)	
No	26 (23)	25 (22)	
**Chemotherapy**			*p* < 0.001
Yes	43 (33)	71 (55)	
No	88 (67)	59 (45)	
**MUST**			*p* = 0.152
0	65 (50)	76 (59)	
1/2	66 (50)	54 (41)	
**Reported Weight Loss**			*p* = 0.751
Yes	60 (46)	57 (44)	
No	71 (54)	73 (56)	
**Low BMI** ^**c**^			*p* = 0.091
Yes	19 (19)	7 (9)	
No	83 (81)	67 (91)	
**Low SMI** ^**d**^			*p* = 0.788
Yes	48 (44)	36 (64)	
No	55 (56)	38 (36)	
**Low SMD** ^**e**^			*p* = 0.338
Yes	37 (65)	32 (49)	
No	64 (35)	41 (51)	
**NLR**			
< 3	43 (33)	32 (25)	*p* = 0.053
3–5	48 (37)	44 (34)	
> 5	40 (30)	54 (41)	
**mGPS**			*p* = 0.029
0	44 (34)	19 (15)	
1	29 (22)	49 (38)	
2	58 (44)	61 (47)	
**3-month survival**			*p* < 0.001
Yes	113 (86)	67 (52)	
No	18 (14)	63 (49)	
**6-month survival**			*p* < 0.001
Yes	81 (62)	43 (33)	
No	50 (38)	87 (67)	
**12-month survival**			*p* < 0.001
Yes	46 (35)	14 (11)	
No	85 (65)	116 (89)	

a-*P*-value is from χ^2^ analysis or linear-by-linear association

b-224 patients had nodal staging

c-176 patients had a documented BMI

d-An SMI was calculated for 177 patients

e-An SMD was calculated for 174 patients

The relationship between LDH, age and 1-year survival is shown in Table 3a. LDH was significantly associated with 1-year survival independent of age (*p* < 0.001). The relationship between LDH, sex and 1-year survival is shown in Table 3b. LDH was significantly associated with 1-year survival independent of sex (*p* < 0.001). The relationship between LDH, ECOG-PS and 1-year survival is shown in Table 3c. LDH was significantly associated with 1-year survival independent of ECOG-PS (*p* < 0.001). The relationship between LDH, metastasis and 1-year survival is shown in Table 3d. LDH was significantly associated with 1-year survival independent of metastasis (*p* < 0.001). The relationship between LDH, nodal disease and 1-year survival is shown in Table 3e. LDH was significantly associated with 1-year survival independent of nodal disease (*p* < 0.001). The relationship between LDH, chemotherapy and 1-year survival is shown in Table 3f. LDH was significantly associated with 1-year survival independent of chemotherapy (*p* < 0.001). The relationship between LDH, MUST and 1-year survival is shown in Table 3g. LDH was significantly associated with 1-year survival independent of MUST (*p* < 0.001). The relationship between LDH, weight loss and 1-year survival is shown in Table 3h. LDH was significantly associated with 1-year survival independent of weight loss (*p* < 0.001). The relationship between LDH, BMI and 1-year survival is shown in Table 3i. LDH was significantly associated with 1-year survival independent of BMI (*p* < 0.001). The relationship between LDH, SMI and 1-year survival is shown in Table 3j. LDH was significantly associated with 1-year survival independent of SMI (*p* < 0.001). The relationship between LDH, SMD and 1-year survival is shown in Table 3k. LDH was significantly associated with 1-year survival independent of SMD (*p* < 0.001). The relationship between LDH, NLR and 1-year survival is shown in Table 3l. LDH was significantly associated with 1-year survival independent of NLR (*p* < 0.001). The relationship between LDH, mGPS and 1-year survival is shown in Table 3m. LDH was significantly associated with 1-year survival independent of mGPS (*p* < 0.001).

**Table 3:** **A**. The relationship between serum lactate dehydrogenase (LDH), age and 1 year survival (*n* = 261). **B**. The relationship between serum LDH, sex and 1 year survival (*n* = 261). **C**. The relationship between serum LDH, Eastern Cooperative Oncology Group – Performance Status (ECOG-PS) and 1 year survival (*n* = 255). **D**. The relationship between serum LDH, metastasis and 1 year survival (*n* = 261). **E**. The relationship between serum LDH, nodal disease and 1 year survival (*n* = 237). **F**. The relationship between serum LDH, chemotherapy and 1 year survival (*n* = 261). **G**. The relationship between serum LDH, Malnutrition Universal Screening Tool (MUST) and 1 year survival (*n* = 261). **H**. The relationship between serum LDH, weight loss and 1 year survival (*n* = 261). **I**. The relationship between serum LDH, low body mass index (BMI) and 1 year survival (*n* = 176). **J.** The relationship between serum LDH, skeletal muscle index (SMI) and 1 year survival (*n* = 198) **k.** The relationship between serum LDH, skeletal muscle density (SMD) and 1 year survival (*n* = 176) **l**. The relationship between serum LDH, neutrophil-lymphocyte ratio (NLR) and 1 year survival (*n* = 176) **m.** The relationship between serum LDH, modified Glasgow Prognostic score (mGPS) and 1 year survival (*n* = 176)

**a.**	LDH < 250 Units/L (*n* = 130)	LDH *≥* 250 Units/L (*n* = 131)	*p*-value
Age < 65	12/35 (34%)	2/43 (5%)	**0.006**
Age 65–74	16/42 (38%)	7/53 (13%)	**0.047**
Age > 74	19/53 (36%)	4/35 (11%)	**< 0.001**
*p*-*value*	0.294	0.592	
**b.**	LDH < 250 Units/L (*n* = 130)	LDH *≥* 250 Units/L (*n* = 131)	*p*-*value*
Male	29/71 (41%)	5/63 (8%)	**< 0.001**
Female	18/59 (31%)	8/68 (12%)	**0.037**
*p*-*value*	0.117	0.341	
**c.**	LDH < 250 Units/L (*n* = 129)	LDH *≥* 250 Units/L (*n* = 126)	*p*-*value*
ECOG-PS 0/1	29/68 (43%)	8/76 (11%)	**< 0.001**
ECOG-PS 2	16/47 (34%)	4/40 (10%)	**0.005**
ECOG-PS 3/4	2/14 (14%)	1/14 (7%)	0.557
*p*-value	0.160	0.623	
***d.***	LDH < 250 Units/L (*n* = 130)	LDH *≥* 250 Units/L (*n* = 131)	*p*-value
M0	28/69 (41%)	8/56 (14%)	**< 0.001**
M *≥* 1	19/61 (31%)	5/75 (7%)	**0.033**
*p*-value	0.077	0.312	
***e.***	LDH < 250 Units/L (*n* = 114)	LDH *≥* 250 Units/L (*n* = 123)	*p*-value
N0	10/27 (37%)	4/24 (17%)	0.109
N *≥* 1	27/87 (31%)	9/99 (9%)	**< 0.001**
*p*-value	0.727	0.478	
***f.***	LDH < 250 Units/L (*n* = 130)	LDH *≥* 250 Units/L (*n* = 131)	*p*-value
No chemo	28/87 (32%)	4/60 (7%)	**< 0.001**
Chemo	19/43 (44%)	9/71 (13%)	**0.043**
*p*-value	0.620	0.057	
***g.***	LDH < 250 Units/L (*n* = 130)	LDH *≥* 250 Units/L (*n* = 131)	*p*-value
MUST 0	27/64 (42%)	6/77 (8%)	**< 0.001**
MUST *≥* 1	20/66 (30%)	7/54 (13%)	**0.008**
*p*-value	0.603	0.559	
***h.***	LDH < 250 Units/L (*n* = 134)	LDH *≥* 250 Units/L (*n* = 127)	*p*-value
No Weight Loss	28/70 (40%)	7/74 (9%)	**< 0.001**
Weight Loss	19/60 (32%)	6/57 (11%)	**0.006**
*p*-value	0.503	0.921	
***i.***	LDH < 250 Units/L (*n* = 102)	LDH *≥* 250 Units/L (*n* = 74)	*p*-value
BMI *≥* 20	31/83 (37%)	6/67 (9%)	**< 0.001**
BMI < 20	6/19 (32%)	1/7 (14%)	**0.049**
*p*-value	0.781	0.970	
***j.***	LDH < 250 Units/L (*n* = 103)	LDH *≥* 250 Units/L (*n* = 74)	*p*-value
Normal SMI	21/48 (44%)	1/36 (3%)	**< 0.001**
Low SMI	16/55 (29%)	7/38 (18%)	**0.044**
*p*-value	0.277	0.073	
***k.***	LDH < 250 Units/L(*n* = 101)	LDH *≥* 250 Units/L (*n* = 73)	*p*-value
Normal SMD	15/37 (41%)	5/32 (16%)	**0.018**
Low SMD	22/64 (34%)	3/41 (7%)	**< 0.001**
*p*-*value*	0.727	0.334	
***l.***	LDH < 250 Units/L(*n* = 130)	LDH *≥* 250 Units/L (*n* = 131)	*p*-value
NLR < 3	25/44 (57%)	5/31 (16%)	**< 0.001**
NLR 3–5	15/46 (33%)	4/46 (9%)	**0.008**
NLR > 5	7/40 (18%)	4/54 (7%)	0.349
*p*-value	**< 0.001**	0.490	
***m.***	LDH < 250 Units/L(*n* = 130)	LDH *≥* 250 Units/L (*n* = 130)	*p*-value
mGPS 0	27/43 (63%)	5/20 (25%)	**< 0.001**
mGPS 1	8/29 (28%)	4/48 (8%)	0.232
mGPS 2	12/58 (21%)	4/62 (6%)	**0.038**
*p*-value	**< 0.001**	0.177	

Each cell (n=/%), *P*-value is from χ^2^ or linear-by-linear analysis

The relationship between LCAS (Table 1) and clinicopathological characteristics and survival in patients with advanced lung cancer is shown in Table 4. There was a significant association between LCAS and age (*p* = 0.049), chemotherapy (*p* = 0.047), NLR (*p* < 0.001) and 3-month (*p* < 0.001), 6-month (*p* < 0.001) and 1-year survival (*p* < 0.001).

**Table 4:** The relationship between the laboratory cachexia score (LCAS) and clinicopathological variables, Eastern Cooperative Oncology Group performance status (ECOG-PS), weight loss, malnutrition universal screening tool (MUST) score,, body mass index (BMI), skeletal muscle index (SMI), skeletal muscle density (SMD), disease burden, modified Glasgow prognostic score (mGPS), neutrophil-lymphocyte ratio (NLR), and survival in advanced lung cancer (*n* = 260)

	LCAS 0 (*n* = 43)	LCAS 1 (*n* = 49)	LCAS 2 (*n* = 105)	LCAS 3 (*n* = 63)	*p*-value	
**Age**						*p* = 0.049
< 65	8 (19)	16 (33)	33 (31)	21 (33)		
65–74	12 (28)	23 (47)	35 (33)	25 (40)		
> 74	23 (53)	10 (20)	37 (35)	17 (27)		
**Sex**						*p* = 0.943
Male	22 (51)	24 (49)	56 (53)	31 (49)		
Female	21 (49)	25 (51)	49 (47)	32 (51)		
**ECOG-PS**						*p* = 0.157
0/1	23 (54)	35 (73)	52 (50)	34 (55)		
2	19 (44)	9 (19)	37 (35)	21 (34)		
3/4	1 (2)	4 (8)	16 (15)	7 (11)		
**Metastatic Disease**						*p* = 0.186
Yes	18 (42)	23 (47)	56 (53)	23 (38)		
No	25 (58)	26 (53)	49 (47)	38 (62)		
**Nodal Disease**						*p* = 0.154
Yes	22 (65)	38 (86)	68 (74)	9 (17)		
No	12 (35)	6 (14)	24 (26)	44 (83)		
**Chemotherapy**						*p* = 0.047
Yes	14 (33)	26 (53)	40 (38)	34 (54)		
No	29 (67)	23 (47)	65 (62)	29 (46)		
**MUST**						*p* = 0.662
0	21 (49)	24 (49)	58 (55)	37 (59)		
1/2	22 (51)	25 (51)	47 (45)	26 (41)		
**Reported Weight Loss**						*p* = 0.679
Yes	21 (49)	22 (45)	48 (46)	26 (41)		
No	22 (51)	27 (55)	57 (54)	37 (59)		
**Low BMI**						*p* = 0.492
Yes	5 (15)	8 (22)	9 (13)	4 (10)		
No	28 (85)	28 (78)	58 (87)	36 (90)		
**Low Skeletal Muscle Index**						*p* = 0.226
Yes	21 (60)	20 (59)	32 (47)	20 (50)		
No	14 (40)	14 (41)	36 (53)	20 (50)		
**Low Skeletal Muscle Density**						*p* = 0.323
Yes	19 (56)	19 (54)	42 (64)	25 (64)		
No	15 (44)	16 (44)	24 (36)	14 (36)		
**NLR**						*p* < 0.001
< 3	25 (58)	15 (31)	25 (24)	10 (16)		
3–5	14 (33)	22 (45)	41 (39)	15 (24)		
> 5	4 (9)	12 (25)	39 (37)	38 (60)		
**mGPS**						-
0	43 (100)	20 (41)	-	-		
1	-	29 (59)	48 (46)	-		
2	-	-	57 (554)	63 (100)		
**3-month survival**						*p* < 0.001
Yes	41 (95)	42 (86)	62 (59)	34 (54)		
No	2 (5)	7 (14)	43 (41)	29 (46)		
**6-month survival**						*p* < 0.001
Yes	35 (81)	29 (59)	39 (37)	21 (33)		
No	8 (19)	20 (41)	66 (63)	42 (67)		
**12-month survival**						*p* < 0.001
Yes	27 (63)	13 (27)	16 (15)	4 (6)		
No	16 (37)	36 (73)	89 (85)	59 (94)		

**Fig. 1:**
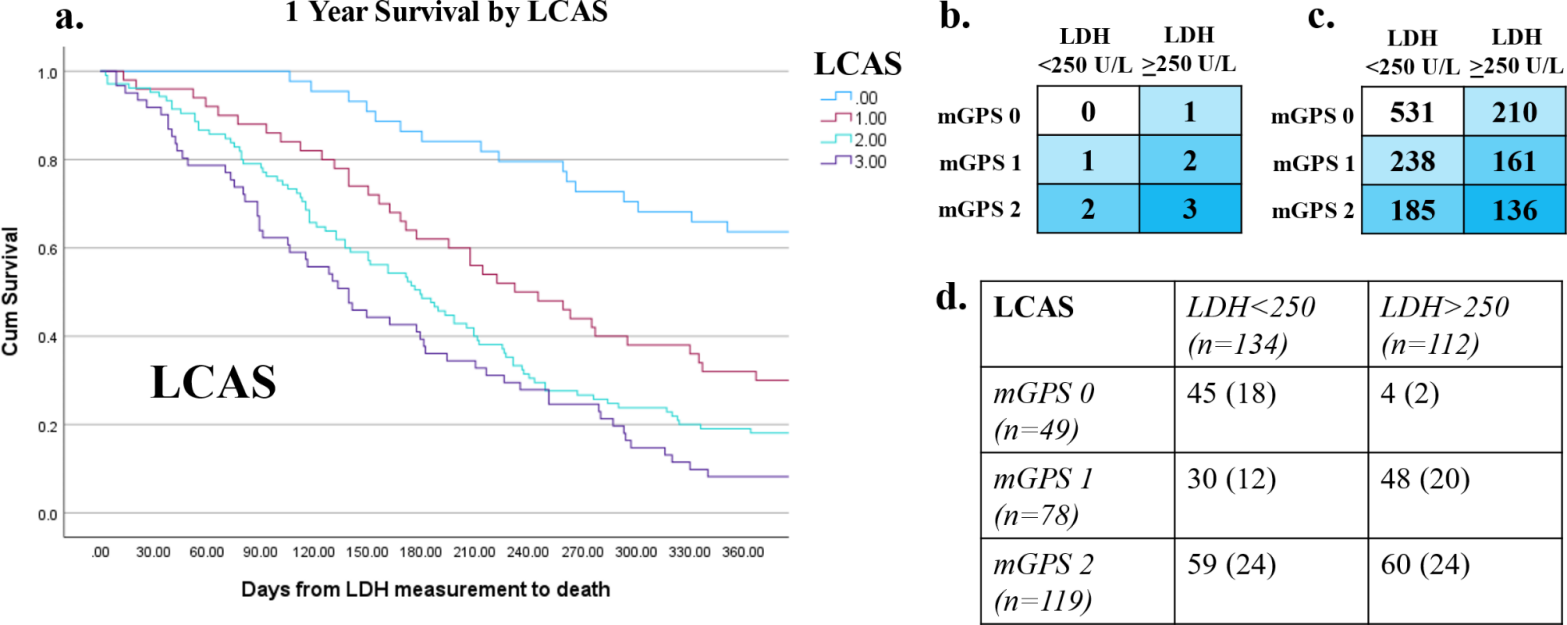
(**a**) Kaplan-Meier demonstrating cumulative survival by laboratory cachexia score (LCAS). Point zero taken as date of lactate dehydrogenase (LDH) measurement. Includes Chi-square value for test of equality of survival distributions for the different categories of LCAS. (**b**) Graphic outlining calculation of the LCAS. (**c**) Median survival in days by LCAS. (**d**) Table outlining the prevalence of each LCAS score within this cohort

**Fig. 2:**
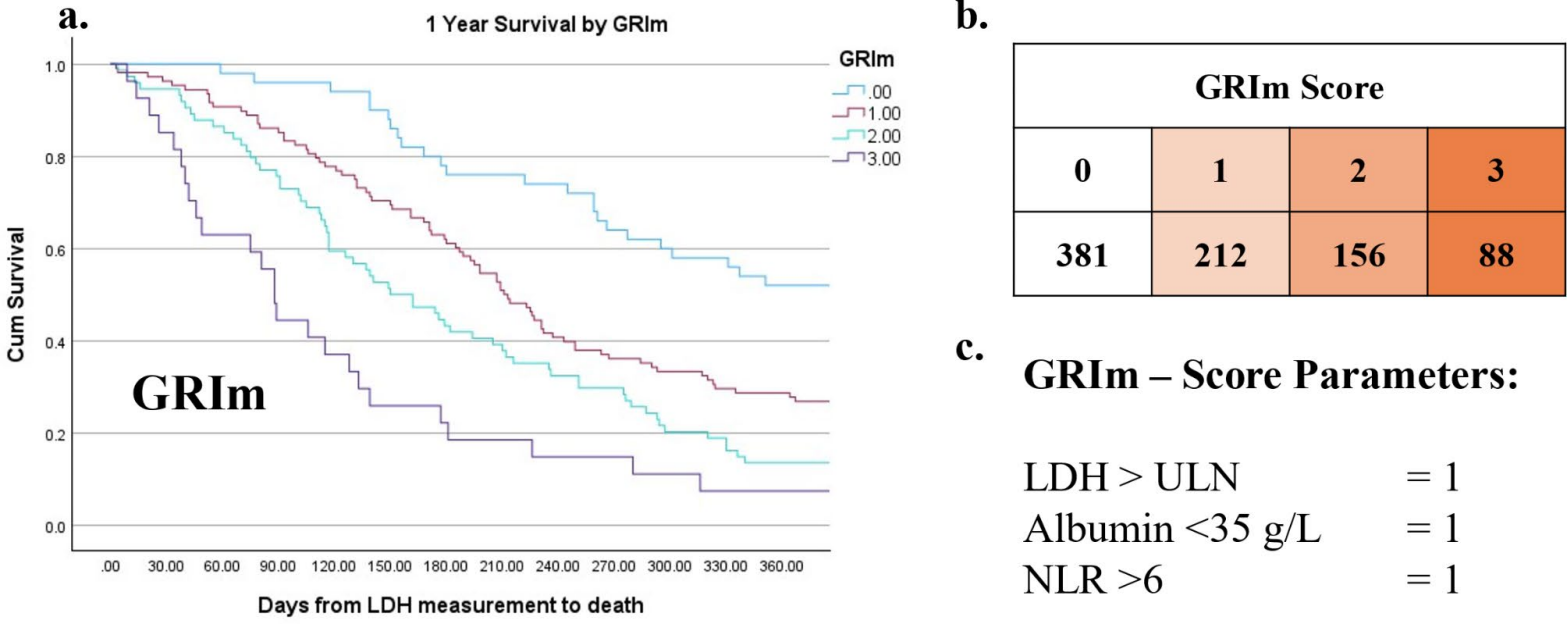
Kaplan-Meier demonstrating cumulative survival by Gustave Rousy Immune (GRIm) – Score. Point zero taken as date of lactate dehydrogenase (LDH) measurement. Includes Chi-square value for test of equality of survival distributions for the different categories of GRIM. b. Median survival in days by GRIm. d. Table outlining the construction of GRIm-Score (0–3)

**Fig. 3:**
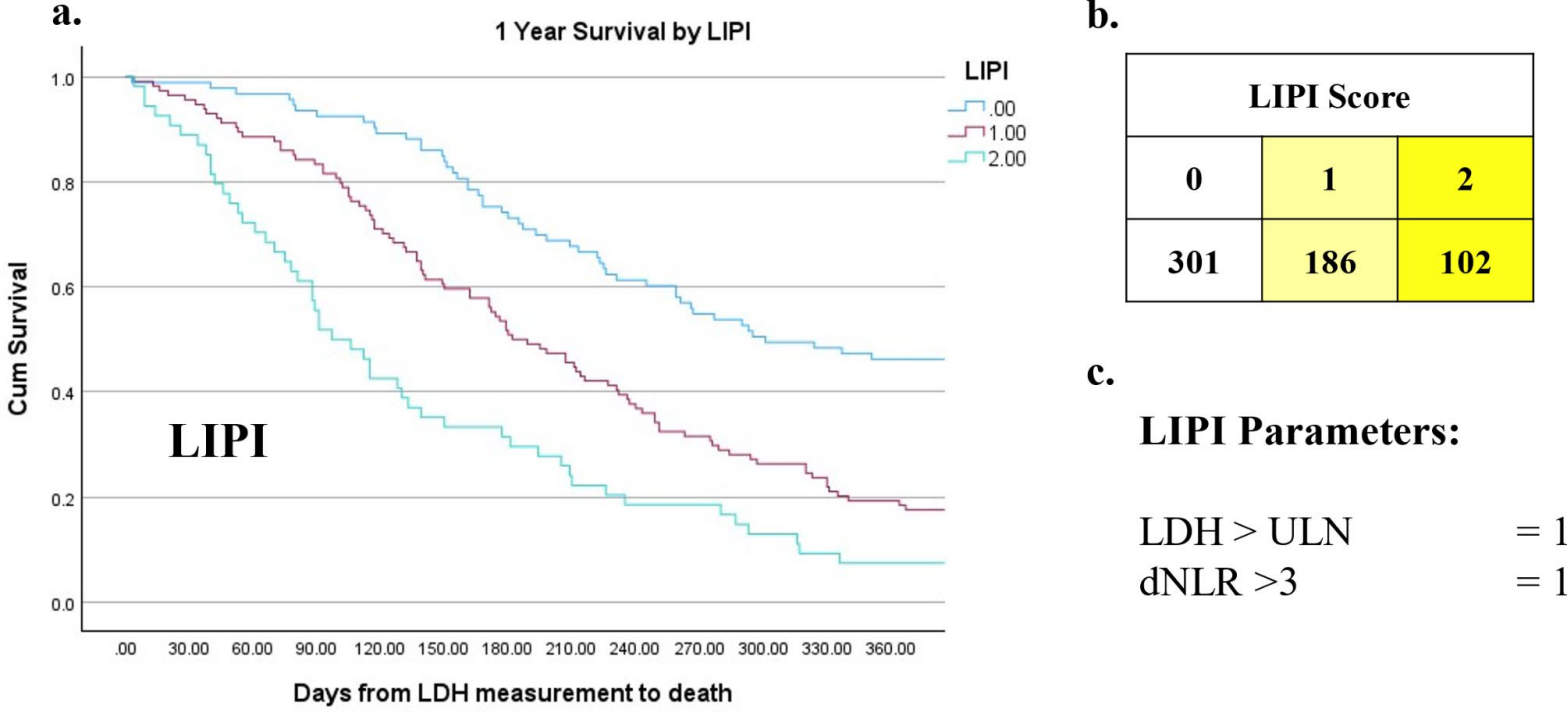
Kaplan-Meier demonstrating cumulative survival by Lung Immune Prognostic Index (LIPI). Point zero taken as date of lactate dehydrogenase (LDH) measurement. Includes Chi-square value for test of equality of survival distributions for the different categories of LIPI. b. Median survival in days by LIPI. d. Table outlining the construction of LIPI (0/1/2 from LDH and derived neutrophil-lymphocyte ratio (dNLR)

The relationship between clinicopathological variables and 1 year survival in patients with advanced lung cancer is shown in Table 5. On univariate analysis metastatic disease (*p* < 0.001), ECOG-PS (*p* = 0.015), NLR (*p* < 0.001), mGPS (*p* < 0.001), LDH (*p* < 0.001), LCAS (*p* < 0.001, Fig. 1), GRIm (*p* < 0.001, Fig. 2) and LIPI (*p* < 0.001, Fig. 3) were significantly associated with 1-year survival. On multivariate analysis (excluding NLR, mGPS and LDH) metastatic disease (*p* = 0.004), ECOG-PS (*p* = 0.019), LCAS (*p* < 0.01, Fig. 1) and LIPI (*p* < 0.01), but not GRIm, remained independently associated with 1-year survival.

**Table 5:** Hazard ratios for overall survival at 1 year from Cox Regression analysis. All variables which were significant on univariable analysis were included in the multivariable Cox Regression

	Univariate Analysis Hazard Ratio (95% CI)	*p*-value	Multivariate Analysis Hazard Ratio (95% CI)	*p*-value
Age (< 65/65–74/>74)	0.85 (0.71–1.01)	0.061		
Sex (male/female)	1.07 (0.81–1.42)	0.638		
Metastatic Disease (yes/no)	1.63 (1.23–2.15)	**< 0.001**	1.53 (1.15–2.04)	**0.004**
ECOG-PS (0–1/2/3–4)	1.29 (1.05–1.59)	**0.015**	1.28 (1.04–1.57)	**0.019**
Chemotherapy (yes/no)	0.86 (0.65–1.13)	0.281		
Weight Loss (yes/no)	1.03 (0.78–1.36)	0.829		
MUST (0 vs. *≥* 1)	1.01 (0.77–1.33)	0.938		
BMI < 20 (yes/no)	1.03 (0.64–1.68)	0.895		
Low SMI	1.03 (0.73–1.45)	0.868		
Low SMD	1.14 (0.80–1.63)	0.478		
NLR (< 3/3–5/>5)	1.66 (1.39–1.99)	**< 0.001**		
mGPS (0/1/2)	1.68 (1.40–2.01)	**< 0.001**		
LDH (*≥* 250 Units/L)	1.93 (1.46–2.56)	**< 0.001**		
LCAS (0/1/2/3)	1.66 (1.43–1.92)	**< 0.001**	1.36 (1.13–1.63)	**0.001**
GRIm (0/1/2)	1.67 (1.43–1.95)	**< 0.001**	0.96 (0.72–1.29)	0.795

The relationship between clinicopathological variables and 1 year survival in patients with non-metastatic lung cancer is shown in Table 6. On univariate analysis NLR (*p* < 0.001), mGPS (*p* < 0.001), LDH (*p* = 0.011), LCAS (*p* < 0.001), GRIm (*p* < 0.001) and LIPI (*p* < 0.001) were significantly associated with 1-year survival. On multivariate analysis (excluding NLR, mGPS and LDH) LCAS (*p* < 0.001), remained independently associated with 1-year survival.

**Table 6:** Hazard ratios for overall survival at 1 year from Cox Regression analysis in patients without metastatic disease (M0). All variables which were significant on univariable analysis were included in the multivariable Cox Regression

	Univariate Analysis Hazard Ratio (95% CI)	*p*-value	Multivariate Analysis Hazard Ratio (95% CI)	*p*-value
Age (< 65/65–74/>74)	0.82 (0.63–1.08)	0.156		
Sex (male/female)	1.14 (0.75–1.74)	0.535		
ECOG-PS (0–1/2/3–4)	1.17 (0.84–1.62)	0.354		
Chemotherapy	1.06 (0.69–1.63)	0.795		
Weight Loss (yes/no)	1.03 (0.67–1.57)	0.894		
MUST (*≥* 1)	1.00 (0.66–1.52)	0.990		
BMI < 20 (yes/no)	1.10 (0.57–2.12)	0.776		
Low SMI	1.47 (0.89–2.44)	0.134		
Low SMD	1.63 (0.94–2.81)	0.080		
NLR (< 3/3–5/>5)	1.79 (1.37–2.35)	**< 0.001**		
mGPS (0/1/2)	2.08 (1.58–2.74)	**< 0.001**		
LDH (*≥* 250 Units/L)	1.73 (1.14–2.62)	**0.011**		
LCAS (0/1/2/3)	1.85 (1.48–2.31)	**< 0.001**	1.85 (1.48–2.31)	**< 0.001**
GRIm (0/1/2)	1.74 (1.36–2.34)	**< 0.001**	0.78 (0.46–1.33)	0.361
LIPI (0/1/2)	1.93 (1.44–2.57)	**< 0.001**	1.28 (0.89–1.84)	0.177

## Discussion

To our knowledge this is the first report to describe a laboratory score of cachexia which combines systemic inflammation as measured by the mGPS with metabolic dysfunction as measured by LDH. In the cohort examined, LDH and mGPS had similar univariate hazard ratios for overall survival (with overlapping confidence intervals) and so could simply be given equal weighting in the construction of new laboratory cachexia score, LCAS. LCAS had prognostic value independent of ECOG, metastatic status and GLIM phenotypic criteria and in patients with non-metastatic disease had most prognostic value, independent of other LDH inclusive scores. Therefore, given that LCAS is based on objective routinely measured clinical values, it has much to commend it for incorporation into clinical practice.

With the realisation of the tumour independent prognostic value of the systemic inflammatory response a plethora of systemic inflammation based prognostic scores and ratios have been developed [24, 26]. However, of these the most established, and extensively used in patients with lung cancer [27, 28], is the modified Glasgow Prognostic Score [29]. The present study is a clear extension of the mGPS with the addition of LDH to the mGPS framework inspired by a prospective study of prognostic factors in patients with advanced cancer [30]. In particular, LCAS compared with other scores including LDH better defined a good prognosis group that may benefit from active treatment (see Fig. 1.). Therefore, with such solid foundations it is likely that LCAS will have clinical utility in patients with advanced cancer and is worthy of external validation.

Interestingly, in patients with non-metastatic disease GRIm and LIPI lost significance in the multivariate survival model whereas the prognostic value of LCAS became stronger. In particular, LCAS compared with other scores including LDH better defined a good prognosis group (Fig. 1.). This may suggest that the inflammatory response represented by the mGPS and the metabolic dysfunction represented by an elevated LDH reflect an environment favorable to tumour progression or prepare the soil for future metastases. Ultimately, the reason for this observation is not clear and future work should examine the prognostic value of LCAS in early-stage lung cancer or those treated with curative intent.

The prognostic value of LDH in patients with advanced cancer has long been recognized and was first incorporated into the Royal Marsden Hospital (RMH) score approximately 15 years ago [31]. More recently, the Gustave Roussy Immune (GRIm), and Lung Immune Prognostic Index (LIPI) scores have included LDH in the presence of NLR. Additionally, the advent of immunotherapy prompted the MD Anderson-Immune Checkpoint Inhibitor (MDA-ICI) [32] and MD Anderson Cancer Centre (MDACC) scores [33]. These LDH focused scores were constructed by retrospectively identifying markers of poor prognosis in phase 1 immunotherapy trials and layering these onto the original RMH score. This results in quite complicated scoring systems. The MDACC adds ECOG-PS and gastrointestinal tumour type, whilst the MDA-ICI requires an LDH > 0.75x ULN and scores six additional parameters (age, ECOG-PS, neutrophil count, lymphocyte count, platelet count and liver metastasis). However, there is a more fundamental concern about the use of the components of a differential white cell count as prognostic scores or ratios, namely that they do not clearly differentiate those patients who are systemically inflamed (as evidenced by CRP) from those that are not [34]. Furthermore, there has been consistent reporting of the superior prognostic value of mGPS compared with NLR [35–38]. Therefore, the use of prognostic scores and ratios based on the components of a differential white cell count should not be preferred over those based on CRP, the prototypical marker of the systemic inflammatory response [39].

Since its introduction in 2003, the Glasgow Prognostic Score has been widely validated in both primary operable cancer [17] and advanced inoperable cancer [40] and in randomized clinical trials [16]. This is in part to its ease of use and consistent prognostic value and therefore it is anticipated that LCAS will be similarly externally validated. It is of interest that recent machine learning models of prognostic factors, in patients with non-small cell lung cancer receiving immunotherapy in the context of a randomized trial, have identified albumin, CRP, LDH and neutrophils as independent prognostic factors [41].

It has become increasingly clear that the tumor-host interaction creates a local and systemic host inflammatory response which drives cancer cachexia. Indeed, inflammation is considered a hallmark of cancer [42]. The basis of the inflammatory response acting as an initiating or driving force in cancer cachexia is not clear. However, it has been postulated that that the interaction of tumour necrosis and local inflammation is an important initiating event [43]. This might explain why the tumour preferentially takes up glucose even in aerobic conditions (the Warburg Effect) using glycolytic substrates and products to fuel tumour growth. In particular, the production of lactate by tumour and host cells will enter the circulation and be transported the liver to be recycled in the lactic acid cycle (i.e. Cori cycle) converting lactate back to pyruvate while also converting NAD + to NADH through the addition of a hydrogen ion. This may result in amplified gluconeogenesis with a reactive hyperinsulinemia which ultimately leads to insulin resistance, a contributing factor to the phenotype of cachexia [44]. Indeed, there is evidence that lactate is pro-tumorigenic promoting angiogenesis, metastasis and tumor resistance with additional roles in immunosuppression and tumor evasion [10, 45]. Irrespective, the present study shows the interaction between LDH and systemic inflammation and their combined prognostic value.

In the present study, when considering the association with 1-year survival, an LDH > 250 Units/L stratified survival in patients who were mGPS 0 and correspondingly mGPS *≥* 1 stratified survival in patients with an LDH < 250 Units/L and survival was significantly worse in patients who had both a raised LDH and mGPS. However, the numbers of observations was relatively small, only 20 of 63 patients (32%) with mGPS 0 in the present cohort had an LDH *≥* 250 Units/L suggesting that metabolic dysfunction is less prevalent in the absence of systemic inflammation. Further work is required in larger cohorts to establish the strength of this relationship.

Interestingly, none of the GLIM phenotypic criteria (unintentional weight loss, low BMI or reduced muscle mass) were associated with either LCAS or overall survival in this cohort of advanced lung cancer patients. In contrast, patients with advanced lung cancer with an LCAS of 0 had 96% survival at 3 months and those with an LCAS of 3 had 54% survival (*p* < 0.001). This suggests LCAS (capturing aetiologic criteria) has superior prognostic value to that of GLIM phenotypic criteria. This has implications for the clinical use of GLIM criteria. Further work is required in larger cohorts to establish the relative prognostic value of aetiologic and phenotypic GLIM criteria.

ECOG-PS has long been the gold standard tool used to stratify the likely outcome of patients with advanced cancer. Although undeniably clinically useful ECOG it is a somewhat subjective measure with the result that outcomes vary even in those patients with good performance status (ECOG 0–1). Additionally, with the advent of immunotherapy the importance of performance status as a barrier to treatment has been questioned, with numerous reports of good outcomes in patients with relatively poor performance status [46, 47]. However, Khaki and colleagues [48] recently voiced concerns about the lack of real world patients included in immunotherapy trials, and highlighted evidence showing worse response rates, faster progression and shorter overall survival in immunotherapy patients with an ECOG-PS *≥* 2 [49–52]. They called for a predictive biomarker that could identify poor performance status patients who might benefit. Predicting survival has always proven challenging in the recruitment of phase I trials of advanced cancer and cachexia treatments, as a survival of at least 3 months is required to obtain the necessary outcomes. Whilst the experienced clinician may be able to identify the dying patient, end of the bed assessments and tools such as ECOG-PS contain an element of subjective judgement [53]. Simple and objective prognostic scores such as LCAS which can stratify survival at 3, 6 and 12 months may prove useful in trial recruitment. Additionally, LCAS may augment decision making in the clinic concerning issues such as transition from systemic therapy to symptom control and discussions around disease trajectory.

In this context objective markers such as LCAS may become increasingly useful. Indeed, recent work published by Saal and colleagues would support such an approach [27]. They reported that patients with radiological evidence of disease progression but a low risk mGPS had a better outcome that patients with stable disease but a high risk mGPS. These results make the argument for routine and serial measurement of mGPS in trial patients. It remains to be determined whether LCAS will be a further improvement to objective monitoring.

There are several limitations to the present study to assess the clinical utility of a novel prognostic score. Firstly, the retrospective nature of this single center work may introduce selection bias and greater than 90% of subjects had a non-small cell lung cancer subtype. However, given the simplicity of the approach used (the use of routinely available laboratory measures) prospective external validation of the present findings should be readily carried out. Lastly, the present study pre-dates the era of immunotherapy, which has fundamentally changed the management of advanced lung cancer and so it would be of particular interest to externally validate LCAS in a population who have received immunotherapy and within different lung histopathological subtypes.

In conclusion, LCAS is an objective measure of inflammation and metabolic dysfunction which effectively identifies cachexia and stratifies survival in patients with advanced lung cancer. Although the underlying mechanisms remain undefined, LCAS may represent a useful addition to the GLIM criteria and a tool for stratifying and monitoring patients enrolled in clinical trials.

## Data Availability

Data is held by the corresponding author and can be made available upon request.

## References

[1] Fearon K, et al. Definition and classification of cancer cachexia: an international consensus. Lancet Oncol. 2011;12(5):489–95. 21296615 10.1016/S1470-2045(10)70218-7

[2] Evans WJ, et al. Cachexia: a new definition. Clin Nutr. 2008;27(6):793–9. 18718696 10.1016/j.clnu.2008.06.013

[3] Cederholm T, et al. GLIM criteria for the diagnosis of malnutrition - A consensus report from the global clinical nutrition community. Clin Nutr. 2019;38(1):1–9. 30181091 10.1016/j.clnu.2018.08.002

[4] McMillan DC. An inflammation-based prognostic score and its role in the nutrition-based management of patients with cancer. Proc Nutr Soc. 2008;67(3):257–62. 18452641 10.1017/S0029665108007131

[5] Yule MS, et al. Biomarker endpoints in cancer cachexia clinical trials: systematic review 5 of the cachexia endpoint series. J Cachexia Sarcopenia Muscle. 2024;15(3):853–67. 38783477 10.1002/jcsm.13491 PMC11154797

[6] Zhang X, et al. A promising prognostic grading system incorporating weight loss and inflammation in patients with advanced cancer. J Cachexia Sarcopenia Muscle. 2023;14(6):2969–80. 37985353 10.1002/jcsm.13376 PMC10751406

[7] McGovern J et al. Are CT-Derived muscle measurements Prognostic, Independent of systemic inflammation, in good performance status patients with Advanced Cancer? Cancers (Basel), 2023. 15(13). 10.3390/cancers15133497 PMC10340321 37444607

[8] Hacker UT, et al. Modified Glasgow prognostic score (mGPS) is correlated with Sarcopenia and dominates the prognostic role of baseline body composition parameters in advanced gastric and esophagogastric junction cancer patients undergoing first-line treatment from the phase III EXPAND trial. Ann Oncol. 2022;33(7):685–92. 35395383 10.1016/j.annonc.2022.03.274

[9] McGovern J, et al. Cancer cachexia: a nutritional or a systemic inflammatory syndrome? Br J Cancer. 2022;127(3):379–82. 35523879 10.1038/s41416-022-01826-2 PMC9073809

[10] Claps G, et al. The multiple roles of LDH in cancer. Nat Rev Clin Oncol. 2022;19(12):749–62. 36207413 10.1038/s41571-022-00686-2

[11] Farhana A, Lappin SL. Biochemistry, Lactate Dehydrogenase, in StatPearls. 2023: Treasure Island (FL) ineligible companies. Disclosure: Sarah Lappin declares no relevant financial relationships with ineligible companies.

[12] Walenta S, Voelxen NF, Mueller-Klieser W. Lactate-An Integrative Mirror of Cancer Metabolism. Recent Results Cancer Res. 2016;207:23–37. 27557533 10.1007/978-3-319-42118-6_2

[13] Warburg O. [Origin of cancer cells]. Oncologia. 1956;9(2):75–83. 13335077

[14] McGovern J, et al. Lactate dehydrogenase: relationship with the diagnostic GLIM criterion for cachexia in patients with advanced cancer. Br J Cancer. 2023;128(5):760–5. 36517550 10.1038/s41416-022-02099-5 PMC9977728

[15] Thompson JJ, et al. The relationship between LDH and GLIM criteria for cancer cachexia: systematic review and meta-analysis. Crit Rev Oncol Hematol. 2024;199:104378. 38754770 10.1016/j.critrevonc.2024.104378

[16] Dolan RD, et al. The prognostic value of the systemic inflammatory response in randomised clinical trials in cancer: a systematic review. Crit Rev Oncol Hematol. 2018;132:130–7. 30447918 10.1016/j.critrevonc.2018.09.016

[17] Dolan RD, et al. The role of the systemic inflammatory response in predicting outcomes in patients with operable cancer: systematic review and meta-analysis. Sci Rep. 2017;7(1):16717. 29196718 10.1038/s41598-017-16955-5 PMC5711862

[18] Dolan RD, et al. The prognostic value of systemic inflammation in patients undergoing surgery for colon cancer: comparison of composite ratios and cumulative scores. Br J Cancer. 2018;119(1):40–51. 29789606 10.1038/s41416-018-0095-9 PMC6035216

[19] Vandenbroucke JP, et al. Strengthening the reporting of Observational studies in Epidemiology (STROBE): explanation and elaboration. Ann Intern Med. 2007;147(8):W163–94. 17938389 10.7326/0003-4819-147-8-200710160-00010-w1

[20] Elia M. The ‘MUST’report. Nutritional screening of adults: a multidisciplinary responsibility, 2003.

[21] Oken MM, et al. Toxicity and response criteria of the Eastern Cooperative Oncology Group. Am J Clin Oncol. 1982;5(6):649–55. 7165009

[22] Bigot F, et al. Prospective validation of a prognostic score for patients in immunotherapy phase I trials: the Gustave Roussy Immune score (GRIm-Score). Eur J Cancer. 2017;84:212–8. 28826074 10.1016/j.ejca.2017.07.027

[23] Mezquita L, et al. Association of the lung Immune Prognostic Index with Immune checkpoint inhibitor outcomes in patients with Advanced Non-small Cell Lung Cancer. JAMA Oncol. 2018;4(3):351–7. 29327044 10.1001/jamaoncol.2017.4771 PMC5885829

[24] Dolan RD, et al. A comparison of the prognostic value of composite ratios and cumulative scores in patients with operable rectal cancer. Sci Rep. 2020;10(1):17965. 33087753 10.1038/s41598-020-73909-0 PMC7578034

[25] Martin L, et al. Cancer cachexia in the age of obesity: skeletal muscle depletion is a powerful prognostic factor, independent of body mass index. J Clin Oncol. 2013;31(12):1539–47. 23530101 10.1200/JCO.2012.45.2722

[26] Dolan RD, McMillan DC. The prevalence of cancer associated systemic inflammation: implications of prognostic studies using the Glasgow Prognostic score. Crit Rev Oncol Hematol. 2020;150:102962. 32344318 10.1016/j.critrevonc.2020.102962

[27] Saal J, et al. Integration of on-treatment modified Glasgow prognostic score (mGPS) to improve imaging-based prediction of outcomes in patients with non-small cell lung cancer on immune checkpoint inhibition. Lung Cancer. 2024;189:107505. 38367405 10.1016/j.lungcan.2024.107505

[28] Klumper N et al. C reactive protein flare predicts response to checkpoint inhibitor treatment in non-small cell lung cancer. J Immunother Cancer, 2022. 10(3). 10.1136/jitc-2021-004024 PMC8928397 35292517

[29] McMillan DC. The systemic inflammation-based Glasgow Prognostic Score: a decade of experience in patients with cancer. Cancer Treat Rev. 2013;39(5):534–40. 22995477 10.1016/j.ctrv.2012.08.003

[30] Simmons C, et al. How long have I got?-A prospective cohort study comparing validated prognostic factors for use in patients with Advanced Cancer. Oncologist. 2019;24(9):e960–7. 30975922 10.1634/theoncologist.2018-0474 PMC6738290

[31] Sahin TK et al. Prognostic significance of the Royal Marsden Hospital (RMH) score in patients with Cancer: a systematic review and Meta-analysis. Cancers (Basel), 2024. 16(10). 10.3390/cancers16101835 PMC11120545 38791914

[32] Sen S, et al. Development of a prognostic scoring system for patients with advanced cancer enrolled in immune checkpoint inhibitor phase 1 clinical trials. Br J Cancer. 2018;118(6):763–9. 29462132 10.1038/bjc.2017.480 PMC5886120

[33] Wheler J, et al. Survival of 1,181 patients in a phase I clinic: the MD Anderson Clinical Center for targeted therapy experience. Clin Cancer Res. 2012;18(10):2922–9. 22452943 10.1158/1078-0432.CCR-11-2217 PMC4176886

[34] McGovern J, et al. The prevalence and prognostic value of systemic inflammation in good performance status patients with advanced, inoperable non-small cell lung cancer receiving palliative radiotherapy: comparison of composite ratios and cumulative scores. Cancer Med. 2024;13(16):e70139. 39164973 10.1002/cam4.70139 PMC11335809

[35] Proctor MJ, et al. A comparison of inflammation-based prognostic scores in patients with cancer. A Glasgow inflammation outcome study. Eur J Cancer. 2011;47(17):2633–41. 21724383 10.1016/j.ejca.2011.03.028

[36] Leitch EF, et al. Comparison of the prognostic value of selected markers of the systemic inflammatory response in patients with colorectal cancer. Br J Cancer. 2007;97(9):1266–70. 17923866 10.1038/sj.bjc.6604027 PMC2360467

[37] Guthrie GJ, et al. Comparison of the prognostic value of longitudinal measurements of systemic inflammation in patients undergoing curative resection of colorectal cancer. Br J Cancer. 2013;109(1):24–8. 23799846 10.1038/bjc.2013.330 PMC3708558

[38] Choi KW, et al. Inflammation-based score (Glasgow prognostic score) as an independent prognostic factor in colorectal cancer patients. Ann Surg Treat Res. 2014;86(6):309–13. 24949322 10.4174/astr.2014.86.6.309 PMC4062449

[39] Gabay C, Kushner I. Acute-phase proteins and other systemic responses to inflammation. N Engl J Med. 1999;340(6):448–54. 9971870 10.1056/NEJM199902113400607

[40] Dolan RD, et al. The role of the systemic inflammatory response in predicting outcomes in patients with advanced inoperable cancer: systematic review and meta-analysis. Crit Rev Oncol Hematol. 2017;116:134–46. 28693795 10.1016/j.critrevonc.2017.06.002

[41] Benzekry S, et al. Predicting Survival in patients with Advanced NSCLC treated with Atezolizumab using pre- and on-treatment prognostic biomarkers. Clin Pharmacol Ther; 2024. 10.1002/cpt.3371 39001619

[42] Hanahan D, Weinberg RA. Hallmarks of cancer: the next generation. Cell. 2011;144(5):646–74. 21376230 10.1016/j.cell.2011.02.013

[43] Vakkila J, Lotze MT. Inflammation and necrosis promote tumour growth. Nat Rev Immunol. 2004;4(8):641–8. 15286730 10.1038/nri1415

[44] Masi T, Patel BM. Altered glucose metabolism and insulin resistance in cancer-induced cachexia: a sweet poison. Pharmacol Rep. 2021;73(1):17–30. 33141425 10.1007/s43440-020-00179-y

[45] Certo M, et al. Lactate modulation of immune responses in inflammatory versus tumour microenvironments. Nat Rev Immunol. 2021;21(3):151–61. 32839570 10.1038/s41577-020-0406-2

[46] Veluswamy R et al. Immunotherapy outcomes in individuals with Non-small Cell Lung Cancer and Poor Performance Status. JNCI Cancer Spectr, 2022. 6(2). 10.1093/jncics/pkac013 PMC8921740 35603847

[47] Ramnaraign BH, et al. Immunotherapy Management in Special Cancer patient populations. JCO Oncol Pract. 2021;17(5):240–5. 33710933 10.1200/OP.20.00996

[48] Khaki AR, Glisch C, Petrillo LA. Immunotherapy in patients with poor performance status: the jury is still out on this Special Population. JCO Oncol Pract. 2021;17(9):583–6. 34297600 10.1200/OP.21.00397

[49] Khaki AR, et al. Immunotherapy-based combination strategies for advanced urothelial cancer: a long quest. Cancer. 2020;126(20):4446–50. 32757318 10.1002/cncr.33068

[50] Petrillo LA, et al. Performance status and end-of-life care among adults with non-small cell lung cancer receiving immune checkpoint inhibitors. Cancer. 2020;126(10):2288–95. 32142165 10.1002/cncr.32782

[51] Sehgal K, et al. Association of Performance Status with Survival in patients with Advanced Non-small Cell Lung Cancer treated with Pembrolizumab Monotherapy. JAMA Netw Open. 2021;4(2):e2037120. 33570575 10.1001/jamanetworkopen.2020.37120 PMC7879233

[52] Friedlaender A, et al. The role of performance status in small-cell Lung Cancer in the era of Immune Checkpoint inhibitors. Clin Lung Cancer. 2020;21(6):e539–43. 32499210 10.1016/j.cllc.2020.04.006

[53] Westgaard A, et al. Prognostic Value of Performance Status, Albumin, and CRP in Last-Line Chemotherapy for pancreatic vs. other gastrointestinal cancers-simple tools Matter. Curr Oncol. 2024;31(9):5462–71. 39330032 10.3390/curroncol31090404 PMC11431321

